# Molecular imaging of multiple sclerosis: from the clinical demand to novel radiotracers

**DOI:** 10.1186/s41181-019-0058-3

**Published:** 2019-04-08

**Authors:** Matteo Bauckneht, Selene Capitanio, Stefano Raffa, Luca Roccatagliata, Matteo Pardini, Caterina Lapucci, Cecilia Marini, Gianmario Sambuceti, Matilde Inglese, Paolo Gallo, Diego Cecchin, Flavio Nobili, Silvia Morbelli

**Affiliations:** 1 Nuclear Medicine Unit, IRCCS Ospedale Policlinico San Martino, Largo R. Benzi 10, 16132 Genoa, Italy; 2 Department of Health Sciences (DISSAL), University of Genova, Genoa, Italy; 3 Neuroradiology, IRCCS Ospedale Policlinico San Martino, Genoa, Italy; 4 Clinical Neurology, Department of Neuroscience (DINOGMI), University of Genoa, Genoa, Italy; 5 Clinica Neurologica, IRCCS Ospedale Policlinico, San Martino, Genoa, Italy; 6 CNR Institute of Molecular Bioimaging and Physiology, Milan, Italy; 7 Multiple Sclerosis Centre of the Veneto Region, Department of Neurosciences DNS, University of Padua, Padua, Italy; 8 Nuclear Medicine Unit, Department of Medicine-DIMED, Padova University Hospital, Padua, Italy; 9 Padua Neuroscience Center, University of Padua, Padua, Italy

**Keywords:** Multiple sclerosis, Positron emission tomography, Neuroinflammation, TSPO, Amyloid, Tumefactive multiple sclerosis

## Abstract

**Background:**

Brain PET imaging with different tracers is mainly clinically used in the field of neurodegenerative diseases and brain tumors. In recent years, the potential usefulness of PET has also gained attention in the field of MS. In fact, MS is a complex disease and several processes can be selected as a target for PET imaging. The use of PET with several different tracers has been mainly evaluated in the research setting to investigate disease pathophysiology (i.e. phenotypes, monitoring of progression) or to explore its use a surrogate end-point in clinical trials.

**Results:**

We have reviewed PET imaging studies in MS in humans and animal models. Tracers have been grouped according to their pathophysiological targets (ie. tracers for myelin kinetic, neuroinflammation, and neurodegeneration). The emerging clinical indication for brain PET imaging in the differential diagnosis of suspected tumefactive demyelinated plaques as well as the clinical potential provided by PET images in view of the recent introduction of PET/MR technology are also addressed.

**Conclusion:**

While several preclinical and fewer clinical studies have shown results, full-scale clinical development programs are needed to translate molecular imaging technologies into a clinical reality that could ideally fit into current precision medicine perspectives.

## Background

Multiple sclerosis (MS) is a progressive immune-mediated inflammatory disease that affects myelinated axons in the central nervous system (CNS), destroying the myelin and the axon in variable degrees and producing physical disability (Reich et al. 2018). MS is among the leading causes of onset of neurological disability in young adulthood, thus representing an enormous social and economic burden in the Western world. MS diagnosis involves a careful medical history, a detailed neurological exam, brain and spine magnetic resonance imaging (MRI), and cerebrospinal fluid analysis (Thompson et al. 2018). In recent years, although MS remains a clinical diagnosis, MRI has become an invaluable tool in understanding and monitoring the disease and is mandatory to confirm the clinical diagnosis (Traboulsee et al. 2016). Various pulse sequences can be used but T2-weighted brain imaging (with emphasis on the spatial and temporal distribution of lesions) remains the standard clinical tool. As detailed in this review, MRI metrics can also be used as an outcome measure in clinical trials.

**Fig. 1:**
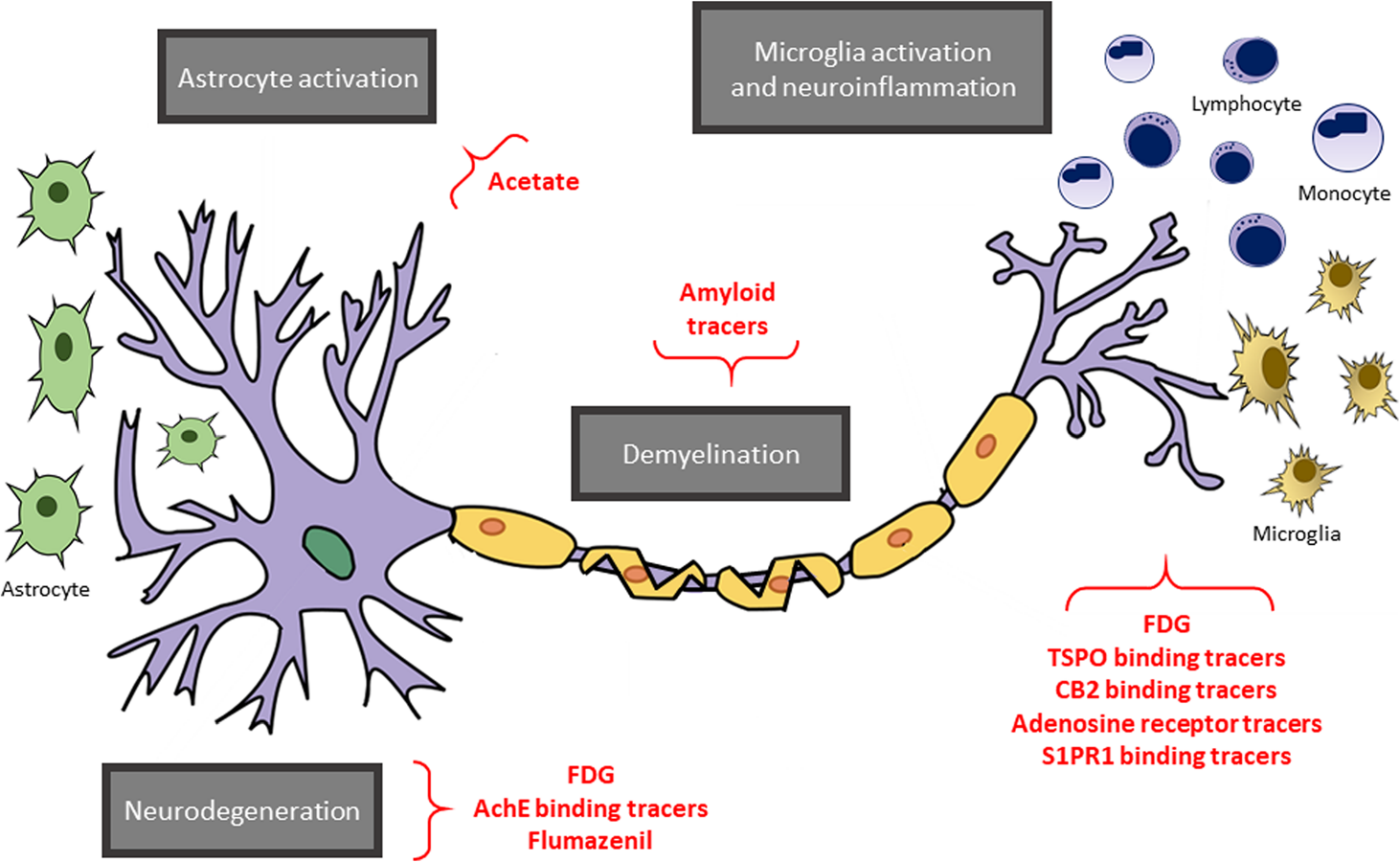
PET imaging targets studied in MS. Main targets of molecular imaging in MS and corresponding PET tracers displaying myelin kinetic, astrocyte activation, microglia activation/neuroinflammation, and neurodegeneration

Positron emission tomography (PET) is a non-invasive technique for quantitative in vivo imaging of biochemical and physiological processes (Magistretti 2000). Brain PET imaging with different tracers is mainly clinically used in the field of neurodegenerative diseases (Morbelli et al. 2017; Bauckneht et al. 2018) and brain tumors (Law et al. 2019). In recent years, the potential usefulness of PET has also gained attention in the field of MS mainly with the aim to fulfil unmet needs in research and drug development. In fact, MS is a complex disease and several processes can be selected as a target for PET imaging. The use of PET with different tracers has been mainly evaluated in the research setting to investigate disease pathophysiology (i.e. phenotypes, monitoring of progression) or to explore its use as a surrogate end-point in clinical trials (De Paula et al. 2014a). The present review deals with PET imaging studies in MS patients and animal models discussing unmet needs in MS drug development and grouping PET tracers according to the different targets (see also Fig. 1). Although to date PET is mainly a research tool in MS, the emerging clinical indication for brain PET imaging in the differential diagnosis of suspected tumefactive demyelinated plaques is discussed. Similarly, the clinical potential provided by PET images according to the current MRI advances as well as in view of the recent introduction of PET/MR technology are also addressed.

## Unmet needs for drug development in MS

In the last decade, a wealth of therapeutic options has been developed by pharmaceutical companies and approved by regulatory agencies to treat people with MS. Currently, more than 15 disease-modifying drugs (DMDs) have been approved to treat MS and others have already shown good potential in phase I-II and ongoing phase III studies (Thompson et al. 2018). Despite the differences in their mechanism of action, all these treatments mainly impact on the inflammatory component of MS, reducing the relapse rates and the formation of new white matter (WM) lesions. MS therapy is thus a success story, at least for tackling the inflammatory phase of relapse-onset MS (Thompson et al. 2018). From a clinical trials perspective, however, two outstanding questions remain regarding novel possible approaches to MS therapy, namely the identification of those progressive MS patients more likely to respond to immunomodulatory therapies (Pardini et al. 2017) and the development of an adequate approach to test for potential neuroprotective and neuro-restorative treatments (Sormani and Pardini 2017).

Regarding the use of immunomodulatory therapies in progressive MS, published data suggest that some progressive patients can present with a clinical benefit from treatment with immunomodulatory drugs (as shown for example in the PPMS ocrelizumab trial or in sub-analyses of studies of other DMDs) (Hawker et al. 2009; Montalban et al. 2017). Identification of those progressive patients with still florid active inflammation, however, is not a trivial task using only the conventional hallmarks of active inflammation such as MRI gadolinium-enhancing lesions; this difficulty led to a restriction of reimbursability of ocrelizumab treatment– and potentially of other DMDs under development as well – in many countries.

MS-related disability, however, is not influenced only by lesion formation and diffuse neuro-inflammation but also by neurodegeneration and by failure of compensatory mechanisms such as remyelination and functional plasticity (Thompson et al. 2018). Recent years have seen a progressive increase in the interest of pharmaceutical companies in the development of neuroprotective and reparative compounds for MS, more so for subjects in the secondary progressive form of the disease, who are currently underserved compared to relapsing remitting patients.

The aim of neuroprotection in MS is to try to reduce the impact of the disease on neuroaxonal loss and to prevent secondary neurodegeneration, whereas reparative treatments aim to mend existing damage by leading to remyelination, restoration of lost synaptic connections, and, ideally, reversal of previous neuroaxonal loss, facilitating endogenous repair processes, or participating directly in tissue repair.

Among those mechanisms of tissue repair, remyelination seems the most achievable soon (and, indeed, some trials of remyelinating compounds have been completed or are underway), but it is not the only possible way for tissue repair.

Remyelination is a dynamic event in MS, and still only partially understood. While in many animal models endogenous remyelination is highly efficient in MS this process is often inadequate, possibly due to a failure of oligodendrocyte cells to populate the MS lesion (Bove and Green 2017). It is mainly evident in newly developed WM lesions, but it is also present also in normal appearing WM and, albeit sporadically, in chronic lesions. Failure of remyelination is thought to represent a factor contributing to development of long-term disability, not only for the less efficient impulse conduction present in demyelinated fibers but also for the neurotropic effect of myelin sheaths on axons (Bove and Green 2017). To date, different approaches to enhance remyelination have been proposed, albeit with mixed results. Interest in the clinical relevance for remyelinating drugs has been recently rekindled by clemastine, which has been shown to improve visual evoked potentials conduction velocity (an indirect neurophysiological marker of myelination) in subjects with demyelinating optic neuritis (Green et al. 2017). The generalizability of these findings outside the optic neuritis model to other brain and cord regions, however, remains a significant challenge for lack of easy-to-use, specific imaging metrics of WM remyelination, that ideally could be used in clinical trials to test the efficacy of remyelinating drugs and possibly in the clinical setting (Sormani and Pardini 2017).

## Value and limits of advanced MRI techniques in MS clinical trials

MRI plays a well-defined role in the diagnosis (Thompson et al. 2018) and follow-up of patients with MS; moreover, it is key in monitoring safety of treatment as it is sensible to early signs of progressive multifocal leukoencephalopathy (PML), a viral opportunistic infection that may affect patients with MS treated with natalizumab (Major et al. 2018). These clinically relevant tasks are accomplished both in clinical routine and in clinical trials by using conventional MRI including T2-weighted, T1-weighted, and post-gadolinium T1-weighted images. These “conventional” sequences are able to identify MS lesions related to “inflammatory” activity and also enable the monitoring of brain parenchyma volume loss (“brain atrophy”) occurring throughout the course of the disease. In particular, disease activity can be radiologically defined by the presence of new or enlarging lesions on T2-weighted images or of enhancing lesions on post-gadolinium T1-weighted images (Río et al. 2017). Since MRI activity is much more frequent than clinical activity, MRI monitoring can be used alongside with neurological evaluation, to better assess response to treatments, today with the aim to achieve “no evidence of disease activity” (NEDA) status. This has an impact on clinical trials which can be designed using conventional MRI endpoints as a correlate of clinical endpoint, thus reducing the study length and the number of patients to enroll (Sormani et al. 2011). Moreover, radiological assessment of inflammatory activity may be relevant for the selection of patients with progressive forms of MS enrolled in clinical trials where drugs with anti-inflammatory activity are tested. Indeed, the impact of drugs with relevant anti-inflammatory activity on disability progression activity is poorer in patients with primary progressive MS (PPMS) without MRI detectable signs of inflammatory activity at baseline with respect to patients presenting MRI features suggestive of ongoing inflammatory activity (Montalban et al. 2017). It is important to underline that, the strong correlation between the effect of immunomodulatory/immunosuppressive drugs on conventional MRI metric and the effect on relapses and clinical disability might not be valid with drugs with a mechanism of action that does not entail anti-inflammatory effects (Pardini et al. 2017; Sormani and Pardini 2017).

An important limitation of conventional MRI is represented by the poor histopathological specificity thus preventing the differentiation between the different features characterizing MS lesions, such as demyelination, neuro-axonal damage, re-myelination and even some aspects of inflammation unrelated to Gd-enhancement, such as smoldering inflammation (Filippi et al. 2019). Indeed, although the degree of T1 hypointensity on T1-weighted imaging has been correlated with the count of residual axons (van Waesberghe et al. 1999), hyperintense signal on T2-weighted images may reflect different histopathological states of MS plaques (e.g. a completely demyelinated, a demyelinated “shadow plaque” and a remyelinated lesion). Consequently, this should be considered when designing clinical trials testing drugs with novel mechanisms of action, for instance molecules aiming at fostering remyelination. In this scenario, more complex MRI metrics useful to perform quantitative analysis should be obtained by using “advanced” MRI techniques. These approaches in the context of multicenter clinical trials, may be difficult to implement, requiring homogenization of MRI protocols among different centres with different technical capabilities and longitudinal quality assessment, in order to obtain reliable results. To quantify myelin content, a few MRI based quantitative methods have been proposed. Myelin water imaging was implemented in the mid-1990s and at that time approximately 25 min were necessary to acquire a single brain slice (MacKay et al. 1994); with newer techniques it is possible to acquire whole brain data in 4 min (Nguyen et al. 2015). Other techniques relatively sensitive to myelin content are represented by Magnetization Transfer Ratio (MTR) imaging, (Chen et al. 2008), although MTR can be influenced by other pathological processes such as oedema. Furthermore, diffusion imaging techniques, in particular acquired with schemes suitable for analysis using multi-compartment models, such as Neurite Orientation Dispersion and Density Imaging (NODDI) (Datta et al. 2017a), can provide data regarding microstructural features of brain tissue with better histological specificity than conventional MRI.

## PET tracers for myelin kinetics

In MS, spontaneous myelin repair occurs, allowing to mitigate the impact of focal and diffuse WM damage, albeit incompletely (Kieseier and Wiendl 2006). Post-mortem evidence suggests that after disease-related demyelination, remyelination can be present across all disease phenotypes both in lesions and in NAWM (Patrikios et al. 2006). In subjects with RRMS, for example, newly formed lesions often present quite a lively amount of remyelination, more so in those lesions which do not progress to black holes (Katz 2015). Remyelination, moreover, is also present in subjects with progressive MS, mainly including PPMS, even in those without MRI evidence of disease activity (Katz 2015). Some drugs have been shown to impact on remyelination of MS damage in clinical trials, even if their translation to clinical practice is still to come (Doshi and Chataway 2017). The development and clinical testing of remyelinating drugs is currently hindered by the lack of quantitative measures able to quantify remyelination across the spectrum of MS using a reproducible approach (Mallik et al. 2014).

To date, no imaging techniques specifically targets demyelination and remyelination. The potential use of PET imaging with tracers for myelin dynamics in MS has gained attention in recent years. The first tracer used for PET imaging of myelin, [^11^C] labeled 1, 4-bis (p-aminostyryl)-2-methoxybenzene (BMB), was evaluated in 2006 in baboons (Stankoff et al. 2006). This tracer showed high affinity for WM, however the presence of poor contrast between GM and WM and the needed long-lasting acquisitions, not feasible given the short half-life of ^11^C, prevented further developments for this tracer (Stankoff et al. 2006). [^11^C] Case Imaging Compound (CIC), a compound with a similar structure as BMB, was also tested for myelin kinetic evaluation in a preclinical study (De Paula et al. 2014d). However, ex vivo data in preclinical models did not confirm the capability of CIC in highlighting remyelination (De Paula et al. 2014d). By contrast, another experimental PET tracer [^11^C]N-methyl-4,4′-diaminostilbene (MeDAS), has shown pharmacokinetics, tissue binding and blood-brain penetration characteristics more favorable for imaging (Wu et al. 2010). MeDAS was demonstrated to selectively label WM regions in the brain and in spinal cord and to have highly sensitivity and specificity to myelin content in experimental models of MS. Finally, it was found to be to decrease in presence of inflammatory demyelination in experimental autoimmune encephalitis and to progressively increase during the subsequent spontaneous remyelination, in agreement with histopathology and clinical symptoms (Wu et al. 2013; De Paula et al. 2014c).

**Fig. 2:**
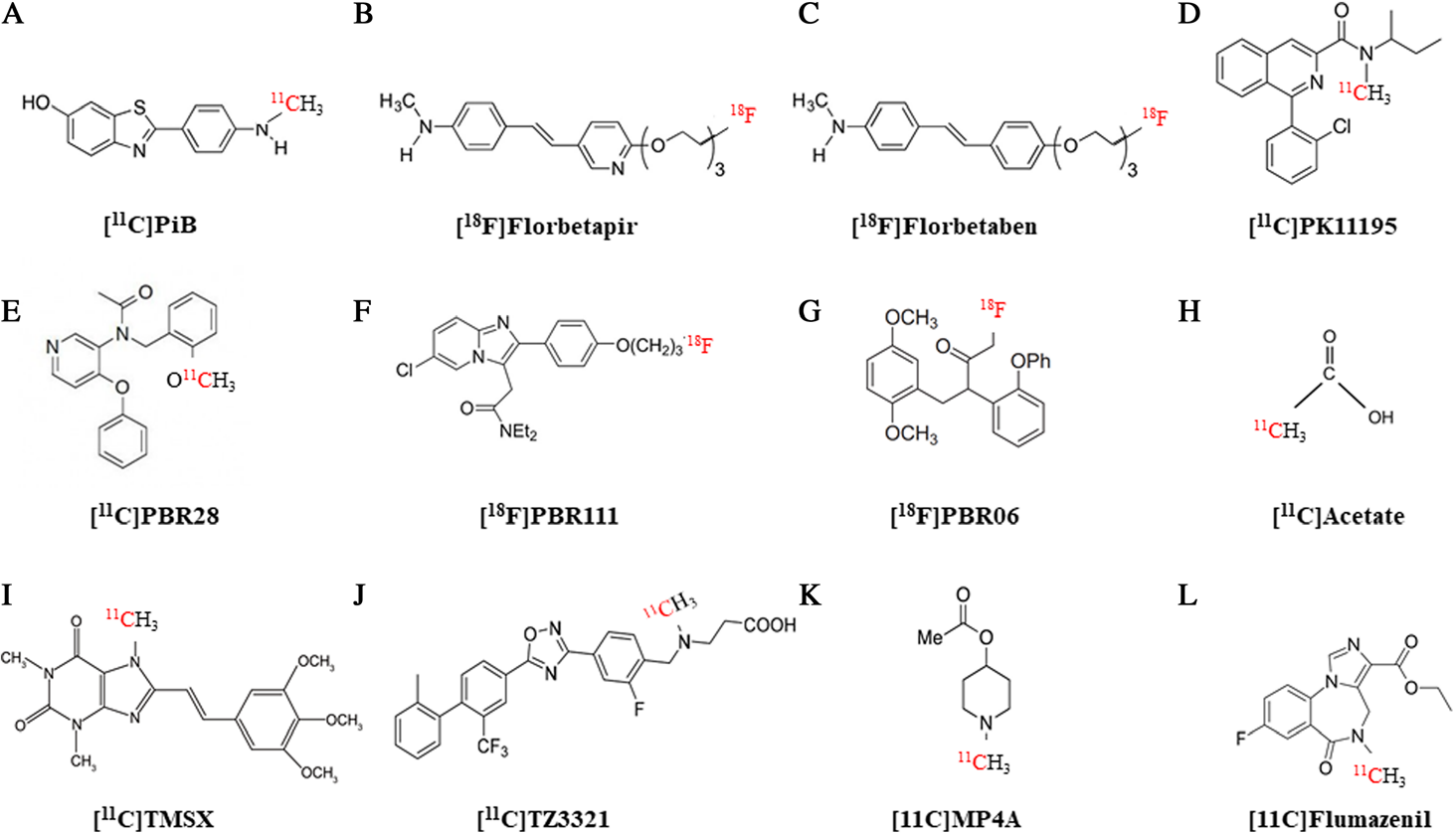
Chemical structures of the most important PET tracers in MS. Chemical structures of the most promising tracers targeting myelin kinetics (Panels **a**-**c**), TSPO (Panels **d**-**g**), astrogliosis (Panel **h**), adenosine receptor (Panel **i**), sphingosine 1-phosphate receptors (Panel **j**) and neurodegeneration (Panels **k**-**l**)

Recently, PET with amyloid tracers, an emerging tool to image amyloid deposition in neurodegenerative diseases, has also been suggested as a potential marker of WM damage in MS (Chandra 2015; Morbelli et al. 2019). All amyloid PET tracers bind to the WM, independently of the presence or absence of beta-amyloid deposition in the adjacent GM (Morbelli and Bauckneht 2018). The high lipid content of the WM and the lipophilic nature of these tracers contribute to their uptake by the WM (Garibotto et al. 2016). The mechanism of this binding is poorly understood. As some Congo red derivatives used to develop amyloid PET tracers show affinity for myelin, it has been suggested that the beta-sheet structure that is present in both the amyloid peptide and in the myelin binding protein (major protein component of myelin) is a common target for amyloid PET tracers (De Paula et al. 2014a). Amyloid PET imaging has been applied to highlight in vivo demyelination in baboons and in small groups of patients with MS and healthy subjects. Several stilbenes and benzothiazole derivatives have been repurposed to image myelin by PET (Bodini et al. 2016). In general, these studies repeatedly demonstrated that amyloid PET detects decreased activity in black hole areas in T1-weighted MR images and in WM lesions in T2-weighted MR images, thus suggesting that amyloid tracer uptake decreases with demyelination (Stankoff et al. 2011). In particular, to date the most used amyloid PET tracer to specifically investigate demyelination in the research settings has been the thioflavine-T derivative 2-(4′-methylaminophenyl)-6-hydroxybenzothiazole labelled with ^11^C (Pittsburgh compound B, PiB, Fig. 2a) (Bodini et al. 2016). In 2011, Stankoff and colleagues studied PiB fixation to myelin by fluorescence in the normal and demyelinating mouse brain, as well as in the postmortem brain of MS patients and demonstrated that demyelinated and remyelinated lesions were clearly distinguishable by the differential intensity of labeling observed with PiB (Stankoff et al. 2011). Following that pilot study, Bodini et al. longitudinally evaluated the capability of PiB to explore myelin dynamics in 20 RRMS patients and in 8 healthy controls. At baseline a progressive reduction in PiB binding from the NAWM to MS lesions (reflecting a decline in myelin content) was demonstrated. They also calculated three dynamic indices for each patient (the global index of myelin content change, the index of demyelination and the index of remyelination) (Bodini et al. 2016). For all indices high between-patient variability of myelin content change was highlighted during follow up and dynamic remyelination was demonstrated to inversely correlate with clinical disability scales. The association of cognitive function in late MS with cortical and WM PiB binding has also been assessed in another group of 24 MS patients identified within the population-based Mayo Clinic Study of Aging (Zeydan et al. 2018). In that study Zeydan and colleagues found no difference in global cortical PiB standardized uptake value ratios between MS and controls. By contrast PiB uptake was lower in areas of WM hyperintensities on MRI compared to NAWM in MS patients and reduced PiB uptake in both the areas of WM hyperintensities and NAWM was associated with decreased visuospatial performance. PiB uptake has also been already compared with MEDAS distribution in a murine model of MS (De Paula et al. 2014b). While both tracers showed uptake and distribution volume in agreement with local myelin status, PiB showed good uptake in the WM in the cerebrum, but low uptake in the cerebellum despite high myelin density in this region. By contrast MeDAS distribution correlated well with myelin density in different brain regions thus suggesting more favorable properties for quantitative PET imaging of demyelinated and remyelinated lesions throughout the CNS (De Paula et al. 2014b). Furthermore, PiB is not available commercially, as given its short half-life (20 min). However, the current existence of fluorinated amyloid PET tracers (i.e., with their longer 109 min half-life), available commercially and already used in therapeutic trials of AD, makes the use of amyloid PET for imaging a demyelination and remyelination a realistic possibility. To date amyloid PET studies in patients with MS have been carried out with [^18^F] florbetapir and [^18^F] florbetaben (Fig. 2b-c, respectively) (Matías-Guiu et al. 2015; Pietroboni et al. 2019). In the study from Matías-Guiu and colleagues, twelve patients diagnosed with MS and 3 healthy controls underwent studies with MRI and [^18^F]florbetaben-PET imaging. [^18^F] florbetaben uptake was measured in demyelinating plaques (appearing as hyperintense lesions in FLAIR sequences), in NAWM, and in GM (Matías-Guiu et al. 2015). Mean SUV relative to cerebellum was demonstrated to be higher NAWM than in damaged WM and to correlate with severity of disability (Matías-Guiu et al. 2015). Moreover, that study included a heterogeneous group of MS patients (5 with RRMS, 5 with SPMS, and 2 with PPMS) and progressive forms, and showed a more pronounced reduction of uptake in the damaged WM with respect to relapsing-remitting form (Matías-Guiu et al. 2015). Similarly, a study involving PET with [^18^F] Florbetapir aimed to investigate amyloid uptake in damaged and NAWM of MS patients, and to specifically evaluate correlations between CSF β-amyloid1–42 (Aβ) levels, amyloid tracer uptake, and brain volumes (Pietroboni et al. 2019). Again, a lower SUV in damaged WM, compared to NAWM, was demonstrated in all patients and a decreased NAWM-SUV was also observed in patients considered in active phase of disease (Pietroboni et al. 2019). Finally, CSF Aβ concentration was able to predict both NAWM-SUV, further linking the role of β-amyloid to early myelin damage, disease activity and clinical progression.

As a matter of fact, most PET tracers proposed to-date to evaluate demyelination consists of hydrophobic molecules able to bind to myelin proteins. Accordingly, these compounds display a high baseline signal in normal WM and sites of demyelination correspond to area of reduced or absent uptake (which might be not easily detected in case of small areas of demyelination). Recently a different approach has been proposed with the idea of developing a PET tracer that specifically binding to a protein on demyelinated axons, thus providing a positive signal in sites of demyelination (Brugarolas et al. 2018). In presence of demyelination, axonal K^+^ channels become exposed and increase in expression (Coman et al. 2006) and a clinically approved drug for MS, 4-aminopyridine (4-AP), has been developed. This drug binds to, and blocks, these channels reducing the aberrant efflux of K^+^ ions and restoring conduction of demyelinated axons (Bever Jr et al. 1994). A radiolabeled derivative of 4-AP was thus developed as a potential PET tracer for imaging changes in myelination. Using autoradiography Brugarolas et al. demonstrated that 4-AP has higher binding in non-myelinated and demyelinated versus well-myelinated CNS regions (Brugarolas et al. 2018). These authors already described a fluorinated derivative of 4-AP and demonstrated that this tracer can detect demyelination in rodents and non-human primates thus representing a valuable PET tracer for detecting CNS demyelination non-invasively (Brugarolas et al. 2018).

## PET tracers for neuroinflammation in MS

Neuroinflammation is a dynamic and complex adaptive response process involving several cell types and pathways (Singhal et al. 2014). Over the past decade, several neuroinflammatory targets have been identified and their corresponding tracers developed making neuroinflammation’s imaging a promising tool for MS. The greatest potential may lay in the imaging of the dynamic interplay between neuroinflammation and the molecular mechanisms that contributes to the disease progression. The recent findings support several actors involved in the neuroinflammatory process underlying MS: translocator protein 18 (TSPO), cannabinoid and adenosine receptors, astrogliosis and sphingosine 1-phosphate receptors. Each of them offers a great variety of specific target for imaging purposes. By contrast, despite the encouraging evidence (Skaper 2007; Shukuri et al. 2011; Shukuri et al. 2016; Cortes-Salva et al. 2018), cyclooxygenase 1 and 2 binding tracers should be further evaluated before their application in the field of MS.

### TSPO binding tracers

Several pathological studies previously reported the occurrence of microglial activation in various stages and in various locations of MS lesions (Calabrese et al. 2015; Kuhlmann et al. 2017; Kutzelnigg and Lassmann 2014; Peterson et al. 2001 see also Table 1). The presence of activated microglia/macrophages in MS lesions was historically evaluated by monitoring changes in glucose metabolism (Kiferle et al. 2011; Schiepers et al. 1997). Results in patients at different stages of the disease indicate that [^18^F] Fluorodeoxyglucose (FDG) might be able to classify WM lesions as either acute (hypermetabolic) or chronic (hypometabolic) based on the degree of glucose consumption (Buck et al. 2012; Paulesu et al. 1996). FDG PET was also recently proposed to evaluate the inflammation in the spinal cord both in animals and in humans (Buck et al. 2012; Marini et al. 2018). However, the high basal uptake of glucose in the brain represents a major limitation, hampering the usability of FDG as a marker of MS lesions, at least in the clinical setting.

**Table 1:** Sites of microglia activation on MS lesions (Peterson et al. 2001; Kutzelnigg and Lassmann 2014; Calabrese et al. 2015; Kuhlmann et al. 2017) and corresponding potential application of neuroinflammation TSPO-PET tracers

Site	Histopathological features	Corresponding TSPO-PET findings in humans
WM lesions	Active lesions: activated macrophages or microglia throughout the lesions with synchronous myelin destruction. Inactive lesions: lack of microglia or macrophages within the MS lesion. Mixed active/inactive lesions: hypocellular core and activated microglia or macrophages at the lesion border. The so-called “smoldering/slowly expanding lesions” are considered a subtype of mixed lesions and are typical in patients affected by progressive forms of MS. (P) reactive lesions*:* microglial activation prior to the formation of the WM lesions (preactive) or in response to remyelination of older WM lesions (reactive).	Heterogeneous PK11195 uptake pattern within the WM lesions has been described (core versus periphery and lesional vs perilesional (Kaunzner et al. 2019; Datta et al. 2017b; Colasanti et al. 2014)). Lesional and perilesional binding of vinpocetine has been shown to be higher than PK11195 but with small overlap in areas of high uptake between ligands (Park et al. 2015). PK11195, PBR28 and PBR111 uptake pattern has been shown to potentially track disease progression as well response to therapy in RRMS patients (Colasanti et al. 2014; Colasanti et al. 2016; Datta et al. 2017a; Datta et al. 2017b; Datta et al. 2017c; Kaunzner et al. 2017; Kaunzner et al. 2019; Oh et al. 2011; Park et al. 2015).
Non-lesional WM	Activated microglia can be seen in the areas immediately surrounding zones of active demyelination plaques, particularly in progressive forms of MS (periplaque WM, PPWM). This finding is associated with initial myelin loss and apoptosis of oligodendrocytes. In the areas of WM that are distant from plaques, which appear normal on MRI and gross pathology (normal appearing WM, NAWM), diffuse microglial activation and occasionally small microglial nodules has been described.	Increased PK11195 and PBR28 binding has been described in NAWM (Datta et al. 2017b; Rissanen et al. 2014), preceding gadolinium enhancement by 4 weeks (Oh et al. 2011). Enlarging T2 lesion volumes at 1-year MRI follow-up correlated with higher NAWM PBR28 uptake at baseline in RRMS (Datta et al. 2017a).
Lesionals and non-lesionals GM	GM lesions are fewer and usually characterized by lower density of T lymphocytes and microglia or macrophages with respect to WM lesions. However, apoptotic cells are increased, and microglia/macrophages retain an activated morphology irrespective of lesion stage or location of these cells (lesion center versus periphery). These peculiar features of GM lesions might at least partially justify the disproportionately selective GM versus WM atrophy.	Increased non-uniform PK11195 (Politis et al. 2012) and 11C-PBR28 (Herranz et al. 2016) GM binding has been described in MS suggesting the occurrence of neuroinflammation in the cortex. This finding is closely linked to poor clinical outcome and, to a lesser extent, to 7neurodegeneration (Politis et al. 2012; Herranz et al. 2016).

To overcome this limitation the peripheral benzodiazepine receptor, also known as TSPO, has been identified as one of the most promising PET imaging targets for neuroinflammatory events in MS. This protein is part of the mitochondrial permeability transition pore and is only minimally expressed in the healthy human brain. TSPO does not necessarily function as a “receptor” in the conventional sense and it is not exactly known its downstream signaling. However, it has been functionally associated with several physiological processes, while its overexpression has been reported to selectively coincide with the occurrence of the proinflammatory M1-like microglial activation related to neuroinflammation and neuronal injury. However, the practical implications of this finding in humans are still unclear, as the existence of M1 and M2 states of microglial activation in human health and disease has been questioned (Ransohoff 2016).

As detailed in Table 1, heterogeneous TSPO uptake within the WM lesions has been described (i.e. lesional vs perilesional uptake (Colasanti et al. 2014; Datta et al. 2017b) and the level of TSPO binding has been shown to be correlated with clinical disability (Colasanti et al. 2014; Colasanti et al. 2016; Rissanen et al. 2014; Rissanen et al. 2018) and response to therapy (Kaunzner et al. 2017). However, TSPO has been targeted not only within MRI-detected focal inflammatory lesions, but also at the rim of chronic active lesions (core versus periphery uptake). Recently, it has been immunohistochemically demonstrated that lesions with a hyperintense rim on quantitative susceptibility mapping at MRI imaging from both secondary progressive MS (SPMS) and relapse-remitting MS (RRMS) patients demonstrate a higher level of innate immune activation as measured on TSPO-PET imaging (Kaunzner et al. 2019). Kaunzner et al. showed that TSPO+ signal throughout the expansive hyperintense border of rim+ lesions co-localized with iron containing CD68^+^ microglia and macrophages. Above all, the authors found that proportionally more TSPO+ microglia cells were found across both lesion types as compared to TSPO+ astrocytes. This supports the conclusion that an increased TSPO signal among MS lesions is predominantly related to activated microglia/macrophages with respect to astrocytes. TSPO-PET is also sensitive to microglial activation in WM defined non-specifically by its abnormal magnetization transfer signal (Datta et al. 2017a; Datta et al. 2017b; Rissanen et al. 2014; Oh et al. 2011) and to lesional and non-lesional grey matter (GM) involvement (Herranz et al. 2016; Politis et al. 2012). Again, in these settings, TSPO+ was correlated with clinical severity (Politis et al. 2012; Datta et al. 2017a) and might be able to assess the longitudinal change after therapy administration (Kaunzner et al. 2017). Altogether, these studies highlight the potential utility of TSPO-PET in the field of MS which is related to the potential capability of this tracer to track those chronic inflammatory changes that are not detected using conventional MRI methods and to non-invasively define disease-relevant neuropathological heterogeneity within T2 lesions and GM inflammation not otherwise able to be characterized by MRI.

The in vivo visualization of TSPO expression was first accomplished through PET with the isoquinoline carboxamide derivate [^11^C]N-butan-2-yl-1-(2-chlorophenyl)-N-methylisoquinoline-3-carboxamide **(**PK11195, Fig. 2d), a non-benzodiazepine ligand specifically binding to this target. Its use has been limited by the low blood–brain barrier (BBB) penetration, the short half-life due to the binding with ^11^C and the high non-specific binding. Accordingly, TSPO is expressed throughout the brain which means that no region can serve as a reference for quantification of specific tracer binding and that the Standardized Uptake Value (SUV) does not directly reflect specific binding since the signal also contains non-specific radioactivity (Schuitemaker et al. 2007). Therefore, a metabolite corrected arterial input function must be obtained and used for a kinetic model from which binding parameters can be estimated (Plavén-Sigray et al. 2018). However, the mathematical dynamic modeling to quantify PK11195 binding in the CNS is not yet fully validated. Each of the proposed approaches has their advantages and disadvantages, and the presence of distinct analytical approaches confounds comparisons across studies (Turkheimer et al. 2007). Indeed, to identify accurate and robust metrics to monitor the activity of chronic MS lesions is a particularly pressing issue for longitudinal comparison studies particularly in the setting of novel treatment evaluation.

To overcome these limitations, several second and third generation TSPO ligands have been developed. Among these, [^18^F]N-fluoroacetyl-N-(2,5-dimethoxybenzyl)-2-phenoxyaniline, [^11^C]PBR28, [^18^F]PBR111, [^18^F]PBR06, [^11^C]N-(5-fluoro-2-phenoxyphenyl)-N-(2-(2-fluoroethoxy)-5-methoxybenzyl) acetamide (FEDAA1106), [^11^C] vinpocetine and [^18^F] Flutriciclamide (GE180) have already been studied in humans in the setting of MS (Airas et al. 2018; Colasanti et al. 2016; Datta et al. 2017a; Datta et al. 2017b; Datta et al. 2017c; Herranz et al. 2016; Owen et al. 2012; Park et al. 2015; Takano et al. 2013; Vas et al. 2008; Vomacka et al. 2017; Venneti et al. 2013; Table 1). Among them, PBR28 (Fig. 2e) is the most widely studied. Its main advantage is related to the improved signal to background ratio (Owen et al. 2014). However, PBR28 suffers from an unexpected low binding status in over 30% of the population, which complicates the interpretation of the results of published studies and, more importantly, limits its use in the clinical setting, demanding genetic testing for the TSPO polymorphism prior to neuroinflammation-PET imaging. Indeed, a single nucleotide polymorphism in exon 4 of TSPO gene (rs6971) has been identified as the major determinant of affinity binding between TSPO and PBR28 (Owen et al. 2012). Moreover, the binding with ^11^C reduces its practical use, demanding the availability of an on-site cyclotron.

However, currently over 80 high-affinity second-generation TSPO tracers are at some stage of development (Venneti et al. 2013; Airas et al. 2018). Among them, [^18^F]PBR111 (Fig. 2f) demonstrated increased hippocampal binding compared with healthy controls that correlated with depression in patients with MS (Colasanti et al. 2016) and increased NAWM binding which inversely correlated with magnetization transfer ratio, suggesting a potential link between microglial activation and demyelination in regions that appear normal on routine T2-weighted images (Colasanti et al. 2014). However, in vitro, ex vivo and in silico data indicate that [^11^C]PBR28 has higher binding affinity for TSPO and higher displaceable to non-displaceable binding compared with [^18^F]PBR111, when matched for TSPO binding status (Guo et al. 2012). [^18^F]PBR06 (Fig. 2g) is another emerging potential alternative to [^11^C]PBR28. Besides the ^18^F labeling, this tracer has been demonstrated to have more than 90% specific binding to TSPO in animals and to have a threefold less variability than PBR28 in tracer binding due to genetic polymorphism (Dickstein et al. 2011). Moreover, when directly compared in humans, [^18^F]PBR06-derived (but not PBR28) SUV_max_ ratio (the normalization of SUV_max_ to global brain radioactivity) correlated with MS clinical severity (Singhal et al. 2018), suggesting an higher clinical relevance for [^18^F]PBR06.

### Cannabinoid receptor 2 binding tracers

Cannabinoid receptors (CB) are G-protein-coupled receptors which can be divided into two subtypes, type 1 (CB1) which is mainly expressed in the CNS and type 2 (CB2) which has been found particularly in the immune system, but also in certain parts of the CNS (Chiurchiù et al. 2018). Differently from CB1, CB2 has been proposed as a potential target of molecular imaging of neuroinflammation due to its overexpression in the CNS following microglial activation. Accordingly, although its role in neuroinflammation is not fully understood yet, CB2 receptor expression is expected to increase 10- to 100-fold under neuroinflammatory conditions, perhaps due to an early neuroprotective response to injury (Fernández-Suárez et al. 2014; Lourbopoulos et al. 2011; Savonenko et al. 2015). Currently, the most promising CB2 binding tracer is 2-oxo-7-[^11^C]-methoxy-8-butyloxy-1,2-dihydroquinoline-3-carboxylic acid cyclohexylamide (NE40). This tracer was initially tested in an animal model with CB2 receptor overexpression (Evens et al. 2012). In humans the biodistribution of NE40 was tested in a single study on healthy volunteers, demonstrating that NE40 is characterized by rapid brain uptake as well as washout in human brain, which might be beneficial for imaging of neuroinflammation (Ahmad et al. 2013). The next logical step will be an evaluation of NE40 in patients with neuroinflammatory disorders such as MS to investigate whether the level of CB2 receptor overexpression in these disorders is sufficient to be detected by PET imaging.

### Molecular imaging of astrogliosis

Astrocytes are not immune cells per-se*.* However, in specific conditions such as neuroinflammation, they can exert both pro- and anti-inflammatory effects on microglia (Min et al. 2006). Indeed, astrocyte proliferation and formation of scars composing a dense network of hypertrophic cells have been proposed as characteristic features of the MS histopathology (Brosnan and Raine 2013).

In the CNS, astrocytes lipid metabolic activity is preferentially based on acetate consumption that is preferentially absorbed by the monocarboxylate transporter. This transporter is increased in the setting of MS (Nijland et al. 2014). Based on these considerations, [^11^C] acetate PET (Fig. 2h) has been proposed as a potential tool to display MS-related astrogliosis in vivo. Takata and colleagues (Takata et al. 2014) showed that [^11^C] acetate SUV was increased in both WM and GM in a series of MS patients compared to healthy controls and demonstrated a significant correlation between [^11^C] acetate uptake and the lesion number in T1- and T2- weighted MRI images. Of note, the strongest correlation was detected between the mean SUV in WM and the T1 black hole number, suggesting that the mean SUV may correlate with axonal damage. Although of interest, to date no other experiences with [^11^C] acetate PET has been reported in literature.

Other selective targets for molecular imaging of the activated astrocytes have also been suggested, including the enzyme monoamine oxidase B or I2-imidazoline receptor. However, their application has not yet been evaluated in the setting of MS, at least in humans.

### Adenosine receptor tracers

Adenosine receptors are G-protein coupled receptors that bind the endogenous neuromodulator adenosine involved in microglial responses. Despite the availability of specific PET tracers for adenosine A1 receptors, such as [^11^C] DPCPX, [^11^C]KF15372, [^11^C] MPDX and the evidence that MPDX PET imaging of the A1R distribution in human brain is feasible (Kimura et al. 2004), to date only studies with A2A-selective radioligand have been published in MS patients. In particular, Rissanen et al. (Rissanen et al. 2013) found an increased A2AR expression in normal-appearing WM of patients with secondary progressive multiple sclerosis detected by [^11^C] TMSX PET (Fig. 2i), directly related to brain tissue loss. Beaino et al. (Beaino et al. 2017) recently demonstrated the potential role of purinergic receptors P2X7R and P2Y12R, expressed within MS active lesions, as promising selective targets for PET imaging of microglia phenotypes in vivo. To date, two radioligands targeting the P2X7 purinergic receptor, namely [^11^C]GSK1482160 (Han et al. 2017) and [^18^F] EFB (Fantoni et al. 2017), have been developed and tested in animal models of neuroinflammation. The main peculiarity of these tracers is the capability of discriminating distinct microglia phenotypes (pro-inflammatory VS anti-inflammatory) in MS, differently from the well-known TSPO tracers.

### Tracers for sphingosine 1-phosphate receptors

Sphingolipids play a crucial role in the nervous system homeostasis. Among them, sphingosine-1-phosphate (S1P) is a cellular membrane sphingolipid able to bind five different subtypes of G protein-coupled receptors (S1PR1–5). These receptors play a major role in the pathophysiology of MS (Choi et al. 2011). It has been shown that S1PR1 expression is significantly increased in MS lesions in postmortem brain tissues (Van Doorn et al. 2010). Moreover, in experimental autoimmune encephalomyelitis, S1P-S1PR1 signaling has been reported to directly activate the differentiation of interleukin 17 producing T cells, which is a key determinant of disease severity in MS (Garris et al. 2013). On the other hand, Fingolimod (FTY-720, Gilenya), the first oral disease-modifying drug approved by the US Food and Drug Administration (FDA) for treating RRMS in 2010 (Kappos et al. 2010), has a marked S1PR1-dependent therapeutic effect on murine experimental autoimmune encephalomyelitis by reducing infiltration of IL-17-producing T cells into the spinal cord (Chiba et al. 2011). Altogether, these data strongly support the involvement of the S1P-S1PR1 signaling in the neuroinflammatory response underlying MS, implying the opportunity to target these molecules by molecular imaging. Several studies previously proposed S1P receptor tracers, including BZM055, W146, and other FTY-720 analogs (Briard et al. 2011; Prasad et al. 2014; Shaikh et al. 2015). However, these ligands either suffer from low rate of phosphorylation or display fast in vivo defluorination, hampering S1P receptor imaging. More recently, Liu et al. optimized the synthesis of [^11^C]TZ3321 (Fig. 2j), which is characterized by a more stable phosphorylation and binds S1PR1 with high selectivity, without binding S1PR2 or S1PR3 (Jin et al. 2017). This tracer showed the capability to detect the increase of S1PR1 expression in rat lumbar spinal cord in an experimental autoimmune encephalomyelitis model of MS. Of note, the increased tracer uptake was paralleled by glial cells and IL-17-producing T cells infiltration (Liu et al. 2016). Obviously, due to the acknowledged limitations related to the ^11^C labeling, several efforts have been also dedicated to the identification of an ^18^F radiotracer for imaging S1PR1 in vivo. To this purpose, Luo and colleagues recently designed and synthesized a fluorinated compound ((1-(4-(5-(4-(2-Fluoroethoxy)-3-(trifluoromethyl)phenyl)-1,2,4-oxadiazol-3-yl)benzyl)-1H-1,2,3-triazol-4-yl)met-hanol), which was evaluated in rodents using ex vivo autoradiography and biodistribution studies that showed good BBB penetration, brain retention and no defluorination (Luo et al. 2018a). More recently, the same group identified other potential ^18^F labeled radiotrecers (Luo et al. 2018b) able to cross BBB and showing high initial brain uptake. However, further validation of obtained tracers in preclinical models of neuroinflammation is needed to identify the most suitable PET radioligand to quantify S1PR1 expression in vivo as a metric of an inflammatory response prior to translation of a lead radiotracer to clinical investigations.

### Other tracers for neuroinflammation

Adjunctive targets for PET imaging have been proposed in the field of neuroinflammation, such as Nitric oxide synthase (Herrero et al. 2012) and folate receptor β (Gent et al. 2013). Similarly, Hoehne et al. (Hoehne et al. 2018) recently showed the potential of a new radiotracer for cystine/glutamate antiporter (xc-), a mediator of glutamate excitotoxicity and proinflammatory immune responses in MS. The authors found a higher sensitivity of this compound -(4S)-4-(3-[18F]fluoropropyl)-l-glutamate (FSPG)- compared to FDG at detecting pathological changes in the spinal cord and brain of mices affected by experimental autoimmune encephalomyelitis. However, they remain in a relatively preliminary stage of development as neuroimaging targets.

## PET tracers for neurodegeneration in MS

Neurodegenerative alterations occur even in the early stages of MS. Chronic neurodegenerative modifications mainly represent the results of the above mentioned neuroinflammatory changes leading to neuronal functional alterations and death. In view of this, a variety of PET radioligands has been used to measure different components of the chronic neurodegenerative process. Actually, [^18^F] FDG, a well-established biomarker in neurodegenerative dementia, has demonstrated to correlate with cognitive impairment also in MS patients (Chiavazza et al. 2018). However, [^18^F] FDG cannot be interpreted as a pure neuronal marker. Indeed, it reflects glucose transport and metabolism within glial cells and neurons in the context of a narrow neuroglial coupling, however as mentioned above, its uptake may also increase in some inflammatory diseases (Marini et al. 2018). These results illustrate the complex interpretation of this PET measure, that reflects both metabolic breakdown and inflammatory burden, justifying the research of PET tracers targeting cholinergic and GABAergic neurotransmission as alternative mark of neurodegeneration in MS.

The axons of cholinergic neurons are often damaged in MS and seems to be involved in the cognitive impairment of MS patients. In fact, acetylcholinesterase (AChE) inhibitors showed a positive response in these patients (Krupp et al. 2004). Based on this consideration, [^11^C]MP4A a tracer able to track AChE activity (Fig. 2k), has been evaluated in patients with MS and dementia. Obtained results surprisingly showed that tracer uptake was inversely related with cognitive impairment probably for an increased AChE activity in the activated microglia (Virta et al. 2011).

Flumazenil is an antagonist of the central benzodiazepine receptor, a component of the ubiquitous GABA-A receptor complex present in the neuronal synapses in the GM. According to the concept that neurodegeneration in GM lesions is likely associated to a loss of GABA A receptors in MS (Rossi et al. 2012), flumazenil can be considered a specific marker of neuronal integrity. Therefore, PET imaging of flumazenil (labelled with ^11^C as in Fig. 2l or with ^18^F) could contribute to assess the neuronal component of MS pathology in vivo. In 2015 Freeman et al. (Freeman et al. 2015) found a reduced [^11^C] flumazenil cortical binding in RRMS and SPMS patients, compared with healthy controls. Intriguingly, GABA A receptor loss was observed independently from the degree of atrophy but well correlated with cognitive performances in enrolled MS patients. Altogether these findings suggest the potential role for this tracer in the identification neuronal damage, which occurs as early as the relapsing-remitting stage, and to localize the cortical regions where neuronal damage predominates. Moreover, in future, neuroprotective and reparative treatment trials that aim to stop or slow down the progression of MS may consider developing the [^11^C] flumazenil cortical binding as outcome measure for clinical trials or to select the patients who show early neurodegenerative changes in GM.

## PET in the differential diagnosis of suspected TMS

TMS is a rare distinct variant of MS posing a diagnostic challenge especially in patients without a pre-existing diagnosis of MS since it might be difficult to differentiate these types of lesions from a CNS neoplasm or from other types of CNS lesions (Algahtani et al. 2017). Accordingly, as clinical, radiological (MRI) and laboratory (CSF) results can be inconclusive, biopsy is often needed to reach a conclusive diagnosis. Given its features, TMS has been previously defined in the literature under several names including demyelinating pseudotumors and tumor-like demyelinating lesions. This variegated nomenclature reflects the tendency of these lesions to mimic a brain tumor clinically, radiologically, and even pathologically (Algahtani et al. 2017). In fact, tumefactive demyelinating lesions (TDLs) are generally defined as acute, large (> 2 cm), tumor-like demyelinating lesions in the CNS occurring with surrounding edema, mass effect, and ring enhancement (Lin et al. 2017). Although there are no pathognomonic imaging signs to indicate a TMS lesion there are characteristics which may be helpful in favoring their identification over a neoplasm or abscess. TDLs are most commonly well circumscribed supratentorial lesions with a predilection for the frontal and parietal lobes, although lesions involving the corpus callosum as well as basal ganglia, infratentorial and spinal cord lesions can occur (Qi et al. 2015). Mass effect and perilesional edema tend to be less evident than those seen with malignancy but increases with larger lesions and those of a more recent onset. The great majority of TMS lesions enhance with gadolinium contrast. While most frequently pathologically-proven tumefactive lesions have a closed ring appearance, almost any pattern of enhancement can be seen (eg, homogeneous, heterogeneous, nodular, punctate, ring) (Kobayashi et al. 2014). In particular, the presence of an open-ring with the open portion joining the GM of the cortex or deep nuclei is suggestive of a demyelinating etiology. Other features typical of a demyelinating etiology include a T2 hypointense rim, peripheral restriction on diffusion weighted imaging and venular enhancement (Hardy and Chataway 2013). Furthermore, TMS lesions tend to have mildly increased diffusion coefficients at MRI diffusion imaging thus helping to distinguish them from abscesses in which diffusion is reduced. However, this aspect is not helpful to tell TMS apart from neoplasm. (Hardy and Chataway 2013). Similarly, MRI perfusion imaging may not be conclusive in some cases due to the variability of cerebral blood volume that characterize different tumor types including glioblastoma multiforme (GBM) and primary CNS lymphoma (PCNSL). For example, PCNSL, being a packed cell tumor, may have decreased cerebral blood volume mimicking TMS (Algahtani et al. 2017).

Moreover, it has been reported that PCNSL can be preceded by demyelinating pseudotumoral brain lesions. The demyelinating lesions recede spontaneously or with corticosteroids and are followed by development of PCNSL (typically within 12 months) thus suggesting the importance of considering PCNSL in patients presenting with a space-occupying lesion, even with previously confirmed demyelination (Ng et al. 2007). Given the challenges related to the differentiation of TMS with respect to neoplastic lesions, the potential role of several PET tracers has been evaluated to support differential diagnosis in this clinical setting (Law et al. 2019). The most widely used PET ligand for imaging in oncology is FDG. However, the role of FDG PET is limited in neuro-oncology due to the high physiological uptake in normal brain GM (Law et al. 2019). Moreover, FDG accumulates in macrophages, making distinction between glioma and inflammatory process difficult. Indeed, increased FDG uptake at TMS lesions (possibly with central hypometabolism) has been already reported thus confirming that FDG PET cannot effectively support the differential diagnosis between neoplastic lesions and TMS (Tarkkonen et al. 2016). In TMS patients submitted to FDG PET other regions of concurrent hypometabolism in structures not directly involved by TLDs has also been highlighted (Chiavazza et al. 2018). While these findings further point out the lack of specificity of FDG PET for the differential diagnosis of TMS, they are consistent with the fact that reduced FDG uptake stands for either a reduction in number of synapses or a reduced synaptic metabolic activity thus possibly providing data on brain functional-metabolic connectivity (Pagani et al. 2017). This finding is of interest as WM lesion and its subsequent damage of cortico-thalamic tracts have been correlated to the so-called “disconnection model” possibly underlying cognitive impairment in MS patients (Patti et al. 2015).

A relatively wider experience for the differential diagnosis of suspected TMS is available for amino-acid (AA) imaging. In fact, based upon increased amino acid uptake of tumor cells in presence of a low background uptake in CNS, radiolabeled amino acids or their analogs has a more established role in neuroncology (Law et al. 2019). To-date, the literature on AA PET and TMS in mainly based on case reports or small case series. In the majority of cases reported in the literature, [^11^C] Methionine (MET-PET) was used to support the differential diagnosis between TMS and neoplastic lesions (Ninomiya et al. 2015; Tarkkonen et al. 2016; Ikeguchi et al. 2018; Yasuda et al. 2018). In these preliminary studies, MET-PET was able to non-invasively diagnose TMS by demonstrating moderate or absent tracer uptake in lesions mimicking GBM on MRI (Ninomiya et al. 2015; Yasuda et al. 2018).

One among the largest case series of TMS has been reported in a study aiming to investigate the utility of proton magnetic resonance spectroscopy (MRS) in differentiating TDLs from gliomas (Ikeguchi et al. 2018). In that study Ikeguchi et al. submitted to MRI imaging and MET-PET, 4 patients with TDLs and 11 patients with high or low grades gliomas. Correlations between MET-PET target-to-normal-tissue ratio (TNR) and each MRS metabolite ratio were examined. Mean Cho/NAA ratio was significantly higher in gliomas than in TDLs and a significant positive correlation was observed between choline/N-acetylaspartate and the MET-PET TNR thus supporting the value of both imaging methods in this clinical setting (Ikeguchi et al. 2018). Choline is a marker of phospholipid turnover in membrane and cellular proliferation, and fiber destruction in gliomas. Previous studies of demyelinating diseases have suggested that increased Cho results from reactive astrogliosis, local ischemia, and neuronal mitochondrial dysfunction (Bolcaen et al. 2013). The use of PET with radiolabeled Choline with ^11^C or ^18^F is also used for differential diagnosis of brain lesion and thus has been reported in a published case of TMS presenting as a pseudotumoral lesion with incomplete ring enhancement, peripheral diffusion restriction, and high choline and lactate peaks on MRS (Bolcaen et al. 2013). Since increased choline turn-over is expected in demyelinating disorders, a similar increased choline uptake should be evident on PET study. However, in this case with pathologically proven TMS, the uptake of [^18^F] choline was very faint and authors hypothesized the presence of a stable plaque with relatively decreased rate of on-going demyelination. (Bolcaen et al. 2013). Similarly, Tarkkonen and colleagues correctly identified by means of both MET and PK11195 a neoplastic lesion (namely a grade II glioma) in a patient with a medical history of MS and other new smaller (T2 hyperintense) demyelinating lesions at cortical (fronto-basal) level and in the spinal cord (Tarkkonen et al. 2016). The suspected (and later on proven) neoplastic lesions was larger with respect to the other and appeared as T1 hypo- and T2 hyperintense without gadolinium-enhancement and again located in the right frontal lobe (Tarkkonen et al. 2016).

**Fig. 3:**
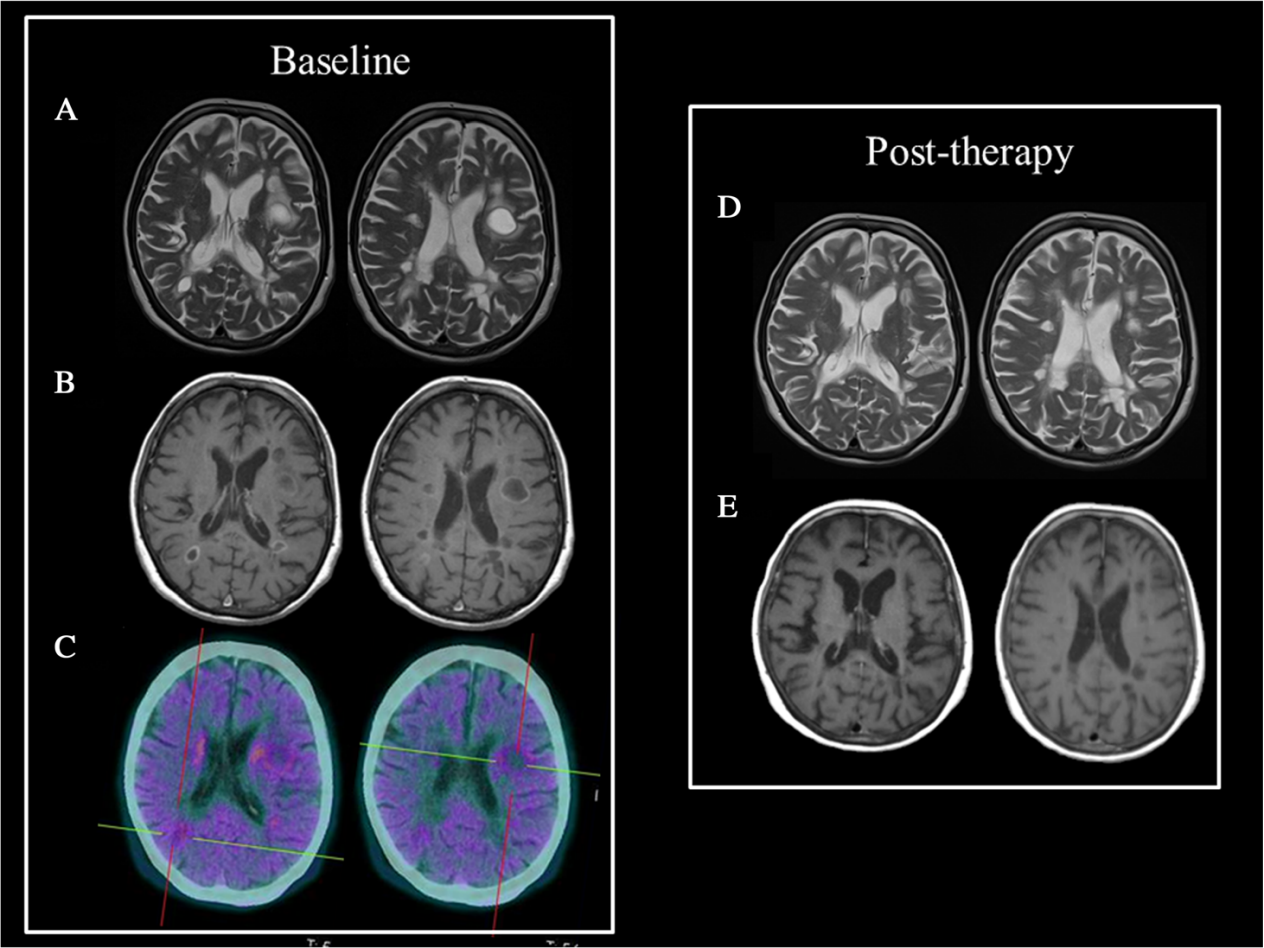
AA-PET and MRI findings in a case of differential diagnosis between TML and breast cancer brain metastasis. 71 years old female patient previously treated for breast cancer who underwent ceMRI that showed the presence of a left frontal lesion hyperintense at T2 sequences (Panel **a**) and hypointense at T1 sequences (Panel **b**). AA-PET/CT with ^18^F-Dopa showed a low tracer uptake supporting the hypothesis of TML. ceMRI performed three months after therapy administration showed the reduction in both lesion dimension (Panel **d**) and enhancement (Panel **e**)

This latter lesion was characterized by significant MET uptake (with a SUV ratio of 2.4 when compared to the contralateral normal tissue) while the other MS lesions were PET negative. By contrast, when evaluated with PK11195, the larger lesion was characterized by very low uptake (within the limits of normal variation) thus further supporting a different nature with respect to an inflammatory/demyelinating lesion (Tarkkonen et al. 2016). Finally, in a patient with a large contrast-enhanced frontal brain lesion, who was initially diagnosed with TMS, [^18^F]fluoroethyl-L-tyrosine (FET), another tracer used for AA-PET, revealed markedly elevated static FET uptake parameters along with time activity-curves consistent with glioma that was later confirmed histologically (anaplastic oligoastrocytoma) (Kebir et al. 2016). As a finale remark, it is worthwhile to underline that, in general, moderate uptake at AA-PET has also been reported in non-tumor CNS diseases (brain abscess, hematoma, radiation necrosis and infarct) possibly implying tracer uptake due to disruption of the blood-brain barrier or to metabolic incorporation due to inflammatory component (Hutterer et al. 2013; Yasuda et al. 2018). Studies in large group of patients are certainly needed to allow the identification of cut-offs of SUV ratio able to accurately differentiate TMS from brain tumors avoiding unnecessary biopsies in these patients. Figure 3 shows example of AA-PET finding in MS together with their corresponding features on MRI.

## Potential of hybrid PET/MR imaging in MS

As already discussed, conventional and advanced MR sequences used to depict spinal cord and WM demyelinating and re-myelinating lesions has made this technique the gold standard to rule out other pathologies (such as neuromyelitis optica or gliomas). However, MR, at present, despite huge advancement in the field (1H-MRS using a number of metabolites (Moccia and Ciccarelli 2017), 23Na MR, MTI (Bodini et al. 2015), DTI, fMRI and others) cannot fully detect the pathophysiological process associated with the disease from the early stages and in particular has limitations in the characterization of the inflammatory status of the WM lesions and in the evaluation of the extent of the pathology in normal appearing white and GM. PET, on the other side, is able to detect microglia activation (using TSPO) or myelin changes (using amyloid tracers) with good accuracy at the cost of a very limited spatial resolution that deteriorates the quantification accuracy especially in small lesions. Recent years have seen a small but growing number of papers leveraging on the unique characteristics of PET/MR scanners to tackle these limitations and improve our understanding of MS physiopathology.

**Fig. 4:**
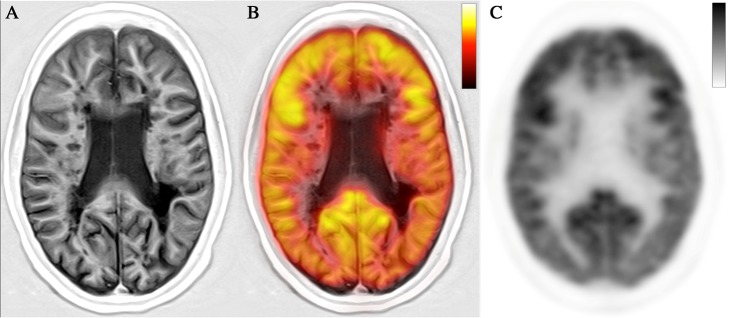
Synchronous PET/MRI acquisition in a RRMS female patient. ^18^F-FDG PET/MRI acquired using Siemens Biograph mMR (University-Hospital of Padova): **a** Phase Sensitive Inversion Recovery (PSIR) sequence showing small multifocal WM lesions in a RRMS female patient. **b** fused ^18^F-FDG PET and PSIR sequence. **c**) ^18^F-FDG PET showing that WM lesions are frequently isometabolic to the WM

Several studies using PET/CT and MR serially, however, have shown that the PET/MR acquisition could offer a number of advantages. In particular, Grecchi et al. (Grecchi et al. 2017) have shown that the application of a multimodal partial volume correction (PVC) of PET using wavelet transform could be successfully applied to MS population overcoming the limitations of classical PVC techniques (the assumption of radiotracer homogeneous distribution within each ROI is clearly inapplicable to MS lesions). The authors were able to obtain high resolution PiB PET with improved quantitative properties and visual quality Derache et al. (Derache et al. 2013), in a cohort of 17 RRMS patients, demonstrated that the complementary results of PET (cerebral metabolic rate of glucose in the basal ganglia) and MR (reduction of GM density in fronto-parieto-temporal areas) could better explain areas involved with the fatigue symptom (affecting up to 80% of MS patients). Datta et al. (Datta et al. 2017c) showed that MRS (of myo-inositol associated with astrocyte activation) and PET PBR28 uptake could be related to distinct inflammatory processes or to elements of a common process with different time courses. Interestingly, because the two markers of brain inflammation can be related under some conditions, in MS patients, the simultaneous acquisition of myo-inositol-MRS and TSPO-PET could potentially show different stages or subtypes of the inflammatory response. The same group (Datta et al. 2017a) demonstrated that the simultaneous evaluation of the glial activation in WM assessed by TSPO-PET (using DVR) and the T2 lesion volume and NAWM magnetization transfer ratio could explain over 90% of the variance in enlarging lesion volume (in relapsing remitting patients) and in greater brain atrophy (in secondary progressive disease) over subsequent 1 year. In all the cited papers the use of an integrated PET/MR system could have been used to further increase patients’ compliance and to improve spatial fidelity and registration accuracy. Furthermore, the synchronous PET/MR acquisition (and not PET/CT and separated MR acquisition) would allow for movement correction of PET data (using MR fast sequences such those used to correct fMRI data) hence reducing blurriness in quantification. This is particularly important when dealing with subtle changes in longitudinal studies. A less obvious but, to us, potentially interesting use of PET/MR in MS will be considering, as recently suggested (Cecchin et al. 2017) that brain physiological processes, including inflammation, and neuronal activity unfold not only in space (where we can use PET/CT + MR with some limitations) but also in time (where only PET/MR is able to depict changes). For instance, one could simultaneously measure specific receptor occupancy or changes due to cold ligands/drugs (e.g. xantinic, benzodiazepine, dintoine and others) vis-à-vis brain network organization (using BOLD or ASL signals) and regional brain blood flow (using [^15^O]H_2_O and ALS). An example of synchronous PET/MR acquisition in a RRMS female patient is reported in Fig. 4.

## Conclusions and future perspectives

The use of PET, even in the research setting, in MS is still in its infancy. However, the relevant number of mechanisms that can be targeted by PET tracers, including myelin repair, innate immune system activation and neurodegeneration might help us in the next future to fill the gap between modern neuroimaging, pathophysiology, neuropathology and drug developments needs in the field of MS. Pilot PET studies performed at different stages of the disease recently showed that patient-specific metrics displaying MS lesion biology could identify trajectories of clinical evolution. Moreover, in the next years, PET studies will presumably guide the development of innovative therapeutic trials aiming to control neuroinflammation and promote remyelination. On the other hand, the potential to combine PET data with MRI findings as a unique non-invasive approach able to display in the same imaging setting different hallmarks of the disease makes the research in this field even more appealing.

## Abbreviations

4-AP: 4-aminopyridine
AA: Amino-acid
AChE: Acetylcholinesterase
Aβ: β-amyloid1–42
BBB: Blood–brain barrier
BMB: 4-bis (p-aminostyryl)-2-methoxybenzene
CB: Cannabinoid receptors
CIC: Case Imaging Compound
CNS: Central nervous system
CSF: Cerebrospinal fluid
DMDs: Disease-modifying drugs
DTI: Diffusion Tensor Imaging
DWI: Diffusion-weighted imaging
FDG: Fluoro-deoxy-glucose
FEDAA1106: N-(5-fluoro-2-phenoxyphenyl)-N-(2-(2-fluoroethoxy)-5-methoxybenzyl) acetamide
FET: 18F-fluoroethyl-L-tyrosine
GBM: Glioblastoma multiforme
GE180: Flutriciclamide
GM: Grey Matter
MeDAS: N-methyl-4,4 ¢-diaminostilbene
MET: Methionine
MRI: Magnetic resonance imaging
MRS: Magnetic resonance spectroscopy
MRT: Magnetization Transfer Ratio
MS: Multiple sclerosis
MTI: Magnetization transfer imaging
NAWM: Normal appearing white matter
NE40: 2-oxo-7-[11C]-methoxy-8butyloxy-1,2-dihydroquinoline-3-carboxylic acid cyclohexylamide
NEDA: No evidence of disease activity
NODDI: Neurite Orientation Dispersion and Density Imaging
PBR: N-fluoroacetyl-N-(2,5-dimethoxybenzyl)-2-phenoxyaniline
PCNSL: Primary CNS lymphoma
PET: Positron emission tomography
PiB: Pittsburgh compound B
PK11195: N-butan-2-yl-1-(2-chlorophenyl)-N-methylisoquinoline-3-carboxamide
PML: Progressive multifocal leukoencephalopathy
PPMS: Primary progressive MS
RRMS: Relapse-remitting multiple sclerosis
SPMS: Secondary progressive MS
SUV: Standardized Uptake Value
TDLs: Tumefactive demyelinating lesions
TMS: Tumefactive multiple sclerosis
TNR: Target-to-normal-tissue ratio
TSPO: Translocator protein 18
WM: White matter
